# The Role of *γδ* T Cells in Systemic Lupus Erythematosus

**DOI:** 10.1155/2016/2932531

**Published:** 2016-02-11

**Authors:** Meng Wu, Jinhua Yang, Xiaofeng Li, Junwei Chen

**Affiliations:** Department of Rheumatology, The Second Hospital of Shanxi Medical University, Shanxi, Taiyuan 030001, China

## Abstract

Systemic lupus erythematosus (SLE) is an autoimmune disease that is characterized by the overproduction of autoantibodies against an array of nuclear and cytoplasmic antigens and affects multiple organs, such as the skin, joints, kidneys, and neuronal tissues. T cells have been recognized as important players in the development of SLE due to their functions in cytokine secretion, antigen presentation, and supporting B cells for antibody production. *γδ* T cells are a minor population of T cells that play important roles in infection and tumor-associated disease. In recent years, the role of *γδ* T cells in autoimmune diseases has been investigated. In this review, we discussed the role of *γδ* T cells in the pathogenesis of SLE.

## 1. Background

Systemic lupus erythematosus (SLE) is a chronic, heterogeneous autoimmune disease that is characterized by the overproduction of antibodies, immune complex deposition, and multiorgan involvement. The pathogenesis of SLE is highly complex, and the breakdown of immunologic self-tolerance is involved. The disorder of T and B lymphocytes plays a vital role in immune dysfunction and mediates tissue inflammation and organ damage. The functions of B lymphocytes in SLE are well described; however, the functional homeostasis of T cell subsets is required for the occurrence, regulation, and maintenance of normal immune responses. Thus, the dysfunction of T cells in SLE should be considered. T cells are divided into two subsets (*αβ* and *γδ* T cells) based on the expression of T cell receptors (TCRs). Unlike *αβ* T cells, *γδ* T cells are a minor population of T cells that consist of *γ* and *δ* chains with a very limited TCR repertoire, recognize primarily nonpeptide antigens, and account for less than 5% of the total T cells in the peripheral blood [1].

Previous studies of *γδ* T cells focused primarily on their anti-infection and antitumor effects, but their functions in the pathogenesis of autoimmune diseases, such as SLE, are not yet well discussed. In this review, we focused on the effect of *γδ* T cells in the context of SLE and provided some insights into the potential roles of these cells in the pathogenesis of this disease.

## 2. Biology of **γ** **δ** T Cells

Since Brenner et al. first discovered and reported *γδ* T cells in 1986 [2], many studies have investigated these cells, from their origin to their functions and their associations with diseases.

*γδ* T cells are a minor population (0.5–5% of total blood) of T cells that carry an alternative TCR heterodimer that is composed of *γ* and *δ* chains. The *γδ* T cell subsets play a crucial role in both the innate and the adaptive immune systems. *γδ* T cells are different from their *αβ* T cell counterparts by using a unique set of somatically rearranged variable (V), diversity (D), joining (J), and constant (C) genes. Moreover, the *αβ* and *γδ* T cell populations recognize different types of antigens. *αβ* T cells recognize non-self-peptide fragments restricted. On the other hand, *γδ* T cells recognize unconventional antigens, including stress molecules, such as MICA and MICB and nonpeptide metabolites of isoprenoid biosynthesis [3–6], among other molecules.

*γδ* T cells exist in the peripheral blood, intestine, skin, spleen, and lymph nodes [7–10] and account for approximately 5–10% of the total T cells [11]. Human *γδ* T cells can be classified into three main groups according to *δ* chain expression: V*δ*1, V*δ*2, and V*δ*3 T cells. V*δ*1 T cells primarily exist in the intraepithelial layer of the skin and intestines, where they are involved in maintaining epithelial tissue integrity when suffering damage, infection, or transformation, responding to stress antigens on epithelial cells and secreting interleukin-10 (IL-10) but little or no interleukin-2 (IL-2), interleukin-4 (IL-4), or interferon-*γ* (IFN-*γ*) [12–15]. V*δ*2 T cells are primarily distributed in the blood and the lymphoid system and constitute the majority of circulating *γδ* T lymphocytes in healthy human individuals, consisting up to 50%–90% of the population of *γδ* T cells in peripheral blood. Most V*δ*2 T cells are V*γ*9*δ*2 T cells, which account for 1–5% of peripheral T cells [16] and are present only in humans and nonhuman primates [17]. Activated V*δ*2 T cells can serve as professional antigen-presenting cells (APCs) [18], such as the expression of antigen-presenting, costimulatory, and adhesion molecules, including major histocompatibility complex (MHC) II, CD80, and CD86 [19]. Furthermore, it is reported that rapamycin or IL-18 treatment can enhance the expression of MHC II, CD80, and CD86 on V*δ*2 T cell lines [19]. V*δ*3 T cells are rare in the blood but rich in the liver and in patients with leukemia and some chronic viral infections, including CD4+, CD8+, and CD4−CD8− subsets. These cells can express CD56, CD161, human leukocyte antigen DR (HLA-DR), and NKG2D but without NKG2A and NKG2C [20]. Upon the stimulation with IL-2, the activated V*δ*3 T cells can recognize CD1d and kill CD1d+ target cells, secrete cytokines such as Th1-, Th2-, and Th17-type cytokines, and induce maturation of dendritic cells (DCs) into APCs [20]. In addition, other populations, such as V*γ*7*δ*4/6, V*γ*4, and V*γ*1, can be found in the intestines and spleen.

Based on the expression of CD27 and CD45RA, *γδ* T cells can also be classified into four subsets: naïve (CD27+CD45RA+), effector memory (CD27−CD45RA−), central memory (CD27+CD45RA−), and terminally differentiated (CD27−CD45RA+) [21].

## 3. Costimulatory Molecules of **γ** **δ** T Cells

The activation of T cells is mediated by two signals: first, TCR recognizes antigens combining with MHC molecules; second, the costimulatory molecules (CD28) combine with the receptor and promote the activation of T cells [22]. Additionally, the negative costimulatory molecules can generate inhibitory cosignals to inhibit the proliferation of T cells. *γδ* T cells can play both positively and negatively regulatory functions through costimulatory molecules and other signal pathways in immune response.

CD28 is an immunoglobulin superfamily receptor that combines with B7.1 (CD80) or B7.2 (CD86) [23], which is expressed on T cells and is known as the most basic costimulatory molecule. Binding to B7 and generating secondary signal, the function of CD28 costimulation in *αβ* T cell activation was well established. However, its relevance to *γδ* T cells has remained controversial [24]. It was observed that CD28 constitutively is expressed on isolated lymphoid *γδ* T cells and plays a positive role in promoting the survival and proliferation of *γδ* T cells in both mice and humans [25]. Moreover, CD28 receptor agonists can significantly enhance the expansion of *γδ* T cells but reversed by B7 antibody-mediated blockade [26]. The major and specific function of CD28 costimulation in *γδ* T cells is to induce the production of IL-2 and IL-2 signals that are of benefit for the expansion of *γδ* T cells [26]. Additionally, the numbers of total or activated *γδ* T cells in CD28-deficient mice are reduced following* Plasmodium berghei* infection. This demonstrates that B7-CD28 costimulatory signals play a vital role in the expansion of *γδ* T cells.

Programmed cell death-1 (PD-1) and B and T lymphocyte attenuator (BTLA) belong to the immunoglobulin (Ig) superfamily and have been well studied recently. PD-1 can bind to PD-L1 (B7-H1; CD274) [27, 28] and PD-L2 (B7-DC; CD273) [27] with different affinities. PD-L2 is three times more potent than PD-L1 in binding to PD-1 [29]. However, the expression of PD-L1 is broader than PD-L2 [29, 30]. The expression of PD-1 on human unstimulated T cells is very low but is induced upon TCR activation. PD-1 exerts an inhibitory effect by inhibiting Akt phosphorylation by interfering with CD28-mediated PI3K activation [29, 31]. Gertner-Dardenne et al. revealed that PD-1 was expressed on resting V*γ*9V*δ*2 T cells and its expression was regulated by the activation of phosphoantigen. Furthermore, Iwasaki et al. found that *γδ* T cells in human peripheral blood expressed PD-1 upon stimulation with nonpeptide antigens and PD-1+ *γδ* T cells produced a significantly higher level of IL-2 in response to an optimal concentration of HMB-PP than PD-1−  *γδ* T cells did [32]. Altogether, these data suggest that PD-1 is an important inhibitory receptor on *γδ* T cells and is also a potential therapeutic target.

BTLA is a recently described member of the CD28:B7 family structurally related to cytotoxic T lymphocyte antigen-4 (CTLA-4) and PD-1. It is expressed on most lymphocytes, including T (*αβ* T cells and *γδ* T cells) and B cells. Its ligand, herpesvirus entry mediator (HVEM), is a member of the tumor necrosis factor (TNF) receptor superfamily expressed on T, B, and NK cells, dendritic cells, and myeloid cells. Binding to HVEM, BTLA can generate negative signals and inhibit the activation and proliferation of T cells [33] and reduce the production of IL-2 and INF-*γ* by T cells. Gertner-Dardenne et al. observed that resting *γδ* T cells expressed a high level of BTLA, particularly on the naïve population. Moreover, the expression of PD-1 was upregulated after TCR engagement, whereas that of BTLA was significantly downmodulated. These results suggest that BTLA and PD-1 may reflect different regulation functions [34].

CD27, one of the TNFR superfamily coreceptors, has also contributed to the activation of T cells. CD27 (TNFRSF7) is expressed on most of V*γ*9V*δ*2 T cells [35] and most of CD27+ V*γ*9V*δ*2 T cells and produces IFN-*γ* with less than 1% of IL-17 with the stimulation of PMA and ionomycin [35]. The proliferation of CD27+ V*γ*9V*δ*2 T cells is sensitive to CD70−CD27 regulation, which generates signals to promote *γδ* T cells activation. CD27 signals can activate the noncanonical NF-*κ*B pathway and promote the expression of antiapoptotic and cell cycle-related genes [36]. Additionally, CD27 costimulation plays vital roles in the protection from activation induced cell death (AICD) following phosphoantigen stimulation [35] and the proliferation of tumor-specific cytotoxic T lymphocytes (CTLs) [37, 38].

NKG2D receptor, a C-type lectin-like receptor, plays important roles in the activation of T cells. It was reported that MICA can activate NKG2D on most human *γδ* T cells [39] and NKG2D can enhance the response of V*γ*9V*δ*2 T cells upon TCR activation.

## 4. Functions of **γ** **δ** T Cells

*γδ* T cells display a number of functions as a primary defense against invading pathogens, especially during early life. These cells can produce an array of cytokines and chemokines [40–42], can regulate the function of other innate and adaptive immune cells, and can also function as APCs [43–46]. The main functions of these cells are detailed below.

First, *γδ* T cells can secrete a variety of cytokines [47] and chemokines to participate in immune responses. Recent studies demonstrated that *γδ* T cells exhibit Th1-, Th2-, Th17-, and Treg-like features [46] and can produce corresponding cytokines, such as the inflammatory cytokines IFN-*γ* and TNF-*α* [48] and the anti-inflammatory cytokines IL-10 [49, 50], TGF-*β* [45], and IL-17, in various infection and autoimmunity models [51–53]. Furthermore, some *γδ* T cells also generate particular cytokines, such as keratinocyte growth factor (KGF) and connective tissue growth factor (CTGF), which play a vital role in the control of epithelial integrity, fibrinogenesis, and wound repair.

Second, *γδ* T cells can function as APCs to recognize MHC and nonprotein phosphoantigens. Brandes et al. [54] showed that activated V*γ*9*δ*2 T cells can express antigen-presenting molecules and costimulatory molecules, such as HLA-DR, CD80, CD86, CD40, and CD54, which are sufficient for the strong induction of the proliferation and differentiation of both naïve CD4+ T cells and naïve CD8+ T cells. In addition, DCs, as an important subset of APCs, can be induced by V*γ*9*δ*2 T cells via TCR-CD1 [55] and Fas-FasL interactions [56]. When V*γ*9*δ*2 T cells are cocultured with immature DCs, the expression of CD86 and MHC class I molecules on iDCs increases significantly [56–58], accompanied by the acquisition of functional activities that are typical of mature DCs [57].

Third, *γδ* T cells can provide a help for B cells [59]. Caccamo et al. found that a subset of CXCR5+ V*γ*9*δ*2 T cells were able to induce a substantial increase in the production of IgG, IgA, and IgM antibodies in the absence of Ag, suggesting that these cells are highly efficient for providing B cell help and play a crucial regulatory role in humoral immunity [60].

Forth, *γδ* T cells can exert immunoregulatory effects. It was well established that regulatory T cells (Tregs) function as negative immune regulation and play an important role in the pathogenesis of several autoimmune diseases. Like *αβ* Tregs, recent studies also found that the existence of a subset of *γδ* T cells with immunoregulatory functions may suppress the activity of CD4+ T cells and DCs [61]. Several studies have revealed that *γδ* T cells in PB are more capable of suppressing the proliferation of CD4+ effector T cells than CD4+ Tregs; backward, V*δ*1 T cells exerted stronger inhibitive activity than V*δ*2 T cells in parallel with increased production of TGF-*β* [62]. Casetti et al. showed firstly that a subset of regulatory V*δ*2 T cells expressing Foxp3 could be induced in vitro in the presence of specific antigen stimulation and cytokines (TGF-*β*1 plus IL-15) [63]. The previous observation also found that *γδ* T cells could exert regulatory functions through Fas/FasL-induced apoptosis of target cells, such as encephalitogenic T cells in various autoimmune diseases [64].

In addition, *γδ* T cells can secrete some cytotoxic components, such as perforin and granzymes, which eventually cause the direct or indirect effect of cytotoxicity [65]. *γδ* T cells also can secrete growth factors such as IGF-1, VEGF, and FGF-2 [12] to maintain epidermal integrity [66].

## 5. **γ** **δ** T Cells in SLE

SLE is an autoimmune disease that is characterized by the enhanced production of autoantibodies and proinflammatory cytokines against a variety of nuclear and cytoplasmic antigens [67] and subsequent damage to multiple organs, such as the skin, joints, kidneys, and neuronal tissues. *γδ* T cells have been demonstrated to play a crucial role in the pathogenesis of autoimmune diseases through their antigen-presenting function, their production of proinflammatory cytokines, their interaction with CD4+CD25+ Tregs, and their promotion of antibody production by providing B cell help [64]. An increased percentage of *γδ* T cells were found in chronic cutaneous lupus erythematosus lesions, with the expansion of the V*γ*2/V*δ*2 subset [68]. We also found that TCR*δ*−/− MRL/lpr mice exhibited exacerbated renal disease and increased mortality, suggesting that *γδ* T cells may be involved in the regulation of lupus [69]. In addition, the population of *γδ* T cells was abnormal in the peripheral blood, skin, and panniculus of SLE patients [70, 71]. Several studies found that *γδ* T cells were present in significantly lower numbers in the PB [72–74] but in higher numbers in the normal cutaneous tissue of SLE patients in comparison to healthy controls [75]. In addition, these cells were associated with SLE disease activity [71]. Some studies found that *γδ* T cells participate in the pathogenesis of SLE. The possible functions of *γδ* T cells in SLE are detailed below.

It is widely recognized that various pro- and anti-inflammatory cytokines, such as IFN-*γ*, IL-4, IL-17, IL-10, and TGF-*α*, play crucial roles in the pathogenesis of SLE [76]. Current studies showed that, in specific microenvironments, *γδ* T cells may divert from the typical Th1-like phenotype and become polarized to Th2 [77, 78], Th17 [78–80], and T regulatory cells [63].

Several studies suggested that SLE is a Th2-driven disease; however, both Th1 and Th2 cytokines are significantly elevated in SLE patients and mice [81]. IFN-*γ* and TNF-*α* have been categorized as Th1 cytokine, while IL-4 and IL-10 have been categorized as a typical Th2 cytokine on pioneering studies. The increasing level of IFN-*γ* may contribute to SLE pathogenesis by inducing B cell activating factor (BAFF) production, MHC I/II expression, and initiating Th1 cell response [76]. Several studies also found that the production of IL-4 and IL-10 was increased in SLE patients. IL-4 can promote the proliferation and differentiation of activated B cells and augment the expression of MHC II molecules. IL-10 can inhibit the proliferation of CD4+ T cells, the secretion of cytokines (such as IL-2, IFN-*γ*, IL-4, IL-5, and TNF-*α*), and apoptosis of T and B cells [76].

Distinct subsets of *γδ* T cells secrete IFN-*γ*, TNF-*α*, IL-10, and IL-4 [64, 82, 83] in a manner similar to Th1 and Th2 cells in response to various pathogens [84]. Some studies showed that, in the peripheral blood of SLE patients, the percentages of *γδ* T cells that expressed intracellular IFN-*γ*, TNF-*α*, IL-10, and IL-4 were all significantly increased, suggesting that different subsets of *γδ* T cells may contribute to the pathogenesis of SLE by secreting pro- and anti-inflammatory cytokines [71, 85].

In contrast to Th1- and Th2-like *γδ* T cells, IL-17-producing *γδ* T cells, which are also known as T*γδ*17 cells, have attracted much attention recently and are known to play important roles in infection, autoimmunity, and antitumor responses. IL-17 can promote T cell activation and infiltration into tissues by upregulating the expression of intercellular adhesion molecule-1 (ICAM-1) [86] and influence B cell proliferation and antibody production [87]. Recent studies validated the role of IL-17 secreted by *γδ* T cells in collagen-induced arthritis (CIA) [40, 88] and rheumatoid arthritis (RA) [89]. However, Lu et al. found that the percentage of *γδ* T cells that express IL-17 was of no significant difference between SLE patients and healthy controls [71], suggesting that IL-17-producing *γδ* T cells may not participate in the pathogenesis of SLE or through another way instead of IL-17 secretion.

As observed for CD4+ Tregs, a subset of *γδ* T cells function as immunoregulatory cells to suppress the activity of CD4+ T cells and dendritic cells [61] via the secretion of TGF-*β* [62]. The regulatory functions of *γδ* T cells have been observed in various autoimmune diseases. Recently, Li et al. found that a subset of CD27+CD25 high V*δ*1 T cells that act as immunoregulatory cells and express Foxp3 were gradually decreased in the PB of SLE patients and exhibited a significant inverse correlation with SLE disease activity, suggesting that these cells can promote the progression of SLE. Furthermore, these regulatory *γδ* T cells could be generated in vitro via *γδ*-TCR stimulation in the presence of IL-2 and TGF-*β* [90].

Several studies showed the enhanced function of regular APCs, including myeloid DCs (mDCs) and monocytes, after the activation of allogeneic T cells in SLE patients [91, 92]. Current data suggest that abnormal APCs functions may be involved in the pathogenesis of SLE because the unusual functions of APCs may downregulate the expression of PD-L1 on their cell surface and result in failed antagonization of T cell signaling transduction mediated by CD80/CD86 and overactivation of effector T cells, thereby leading to lupus onset [93]. *γδ* T cells can express APC-specific molecules, such as HLA-DR and CD80/86, and maintain the activation of CD4+ T cells. Thus, it is possible that the APC-like function of *γδ* T cells may be associated with SLE onset and disease progression via the expression of HLA-DR and CD80/CD86 which may overactivate T cells. More evidence is required to validate this hypothesis.

It is well known that the overactivity and dysfunction of B cells, which lead to the overproduction of autoantibodies, play a vital role in the pathogenesis of SLE. It is well established that T cells play important roles in inducing B cell hyperactivity [94, 95]. Activated T cells can promote immunoglobulin secretion and isotype switching by expressing CD40L [96] and engaging with CD40 on B cells [97]. Moreover, activated T cells can play a major role in costimulating B cells by secreting IL-21 [98, 99]. Studies have also shown that *γδ* T cells can express CD40L and coculture with activated *γδ* T cells resulted in an excessive increase in the B cell-mediated production of IgG, IgA, and IgM [60], suggesting that *γδ* T cells may contribute to a B cell hyperactivity in SLE. In addition, Yin et al. [85] found that CD40L expression levels and IL-21 secretion by *γδ* T cells were significantly elevated in SLE patients. Thus, *γδ* T cells may promote the development of SLE by inducing B cell hyperactivity via the expression of CD40L and secretion of IL-17.

In addition, SLE patients also exhibited decreased levels of the inhibitory receptor NKG2A and increased levels of the activating receptors CD69 and HLA-DR on *γδ* T cells [74].

## 6. Concluding Remarks

The pathogenesis of SLE is elusive, and complex interactions are involved, such as interactions between genetic and environmental factors and interactions between the adaptive and innate immune systems. *γδ* T cells are a unique group of T cells that display a memory phenotype and modulate the function of other innate and adaptive immune cells, function as APCs, and exhibit a Th1-, Th2-, Th17-, and Treg-like phenotype. During the development of SLE, *γδ* T cells play an important role due to the function of antigen-presenting capacity, secretion of proinflammatory cytokines, immunomodulatory properties, interaction with CD4+ Tregs, and ability to promote antibody production by providing B cell help. Additionally, *γδ* T cells are potential targets for cellular immunotherapy, but there still are some obstacles to overcome. Though the evidence of the role of *γδ* T cells in the pathogenesis of SLE remains scarce, further study on their effects in SLE is of great significance for elucidating the pathogenesis of and *γδ* T cells-based therapies for SLE.
